# Bioequivalence of Two Empagliflozin 25 mg Immediate-Release Tablet Formulations Under Fasting Conditions in Healthy Mexican Subjects

**DOI:** 10.3390/ph19060842

**Published:** 2026-05-28

**Authors:** Porfirio de la Cruz Cruz, Alberto Martínez Muñoz, Erika Gabriela Guido Ávila, Omar Emmanuel Hernández Piña, José Trinidad Pérez Urizar

**Affiliations:** 1 Doctorado Institucional en Ingeniería y Ciencia de Materiales (DICIM), de la Universidad Autónoma de San Luis Potosí (UASLP), Av. Sierra Leona No. 550, Col. Lomas 2da. Sección, San Luis Potosí C.P. 78210, Mexico; a365618@alumnos.uaslp.mx; 2 FS SCIENTIA PHARMA S.A. de C.V (Authorized Third Party), Av. Himno Nacional 1330, Jardín, San Luis Potosí C.P. 78270, Mexico; alberto.martinez@fsscientiapharma.com; 3 Asofarma de México S.A. de C.V. Av. Santa Fe 485, Lomas de Santa Fe, Contadero, Cuajimalpa de Morelos, Ciudad de Mexico C.P. 05348, Mexico; eguido@adium.com.mx (E.G.G.Á.); ophernandez@adium.com.mx (O.E.H.P.); 4 Facultad de Ciencias Químicas, de la Universidad Autónoma de San Luis Potosí, Martínez #6, Av. Dr. Manuel Nava, Zona Universitaria, San Luis Potosí C.P. 78210, Mexico

**Keywords:** empagliflozin, bioequivalence, pharmacokinetics, diabetes type 2, SGLT2

## Abstract

**Background/Objectives:** Type 2 diabetes is a group of metabolic disorders whose pathophysiological outcome is sustained hyperglycemia. Several medications are available for the treatment. SGLT2 simultaneously inhibits glucose and sodium reabsorption in the renal proximal tubule, resulting in urinary glucose excretion. This study assessed the pharmacokinetic profiles of two empagliflozin 25 mg drug products under fasting conditions in healthy Mexican subjects to establish bioequivalence. **Methods:** This was a randomized, open-label, two-way crossover, single-dose, prospective study with a 7-day washout period. Eligible subjects were healthy adult Mexican volunteers. The drugs were dosed orally, according to the randomization, after 10 h of fasting and 4 h before breakfast, with 250 mL of 10% glucose solution at room temperature. Serial blood samples were collected before and after dosing. Empagliflozin concentrations were analyzed using high-performance liquid chromatography–tandem mass spectrometry. **Results:** A total of 32 subjects were enrolled, and 30 completed the study. Pharmacokinetic parameters C_max_, t_max_, AUC_0–t_, AUC _0–∞_, and t_½_ of empagliflozin for test and reference formulation, expressed as mean ± SD, were 578.28 ± 125.60 ng/mL, 2.72 ± 0.85 h, 4370.88 ± 769.50 ngh/mL, 4423.93 ± 776.02 ngh/mL, 7.62 ± 0.83 h, and 593.99 ± 156.78 ng/mL, 2.86 ± 1.00 h, 4313.24 ± 885.02 ngh/mL, 4368.04 ± 887.75 ngh/mL, and 7.61 ± 0.68 h, respectively. The 90% CI for C_max_, AUC_0–t_, and AUC _0–∞_ were 98.30 [92.72–104.22], 101.72 [98.77–104.77], and 101.64 [98.73–104.63], respectively. Serious adverse events were not observed. **Conclusions:** Our study demonstrated bioequivalence between the empagliflozin formulations tested in healthy subjects under fasting conditions.

## 1. Introduction

Diabetes mellitus is a group of metabolic disorders of carbohydrate metabolism in which glucose is both underutilized as an energy source and overproduced due to inappropriate gluconeogenesis and glycogenolysis [1]. The pathophysiological outcome is sustained hyperglycemia and its associated complications [2].

Type 2 diabetes (T2D) is caused by a combination of genetic factors related to impaired insulin secretion and insulin resistance, as well as environmental factors such as obesity, lack of exercise, aging, and stress [3]. In 2022, T2D was the second leading cause of death in Mexico, responsible for more than 115,000 deaths. By 2022, 18.5% of the Mexican population had T2D, representing an increase of almost 30% compared to 2006. Furthermore, 32% of people living with T2D were unaware they had the disease. The increase in prevalence and mortality from T2D represents a challenge for Mexico, which faces a rapidly increasing need for screening, care, and treatment [4].

Efficient glycemic control is the cornerstone for reducing the risk of both micro- and macrovascular complications in T2D [5]. The 2025 American Diabetes Association (ADA) guidelines state that the pharmacological management of T2D should address multiple targets, including glucose lowering, weight loss, kidney disease, and cardiovascular disease [6]. Several medications are available for the treatment of T2D. Recently, sodium–glucose cotransporter 2 (SGLT2) inhibitors have gained prominence. These drugs simultaneously inhibit glucose and sodium reabsorption in the renal proximal tubule, resulting in urinary glucose excretion. This group of medications includes canagliflozin, dapagliflozin, and empagliflozin. SGLT2 inhibitors have demonstrated additional benefits in patients with T2D, like promoting weight and blood pressure reduction, decreasing the risk of cardiovascular events, and offering a renal protection effect [7,8,9,10].

Empagliflozin is an SGLT2 inhibitor indicated for the treatment of diabetes, either as monotherapy or in combination [11]. It exhibits high solubility and low permeability in gastrointestinal fluids, classifying it as a BCS (Biopharmaceutical Classification System) Class III drug, and displays linear pharmacokinetics (PK) [12]. Following oral administration, empagliflozin is rapidly absorbed; although absolute bioavailability has not been directly measured, a radiolabel study reported that 75.5–77.4% of plasma radioactivity corresponded to the unchanged parent drug [12,13]. After administering a single 25 mg dose of empagliflozin to healthy Venezuelan subjects under fasting conditions, peak plasma concentrations (C_max_) were observed to occur within a time frame ranging from 1.33 to 3.00 h. The mean peak plasma concentration attained was 339.22 ± 92.14 ng/mL [14].

The decline in plasma concentration following the peak exhibited a biphasic pattern, characterized by an initial rapid distribution phase and a subsequent relatively slow terminal phase. The area under the curve (AUC) for the terminal phase accounted for approximately 16% of the total exposure. Additionally, the maximum plasma concentration (C_max_) represented about 37% when compared to the fasting state [14]. Empagliflozin is distributed into all organs and tissues, [15] with an apparent volume of distribution (Vd) of 73.80 L [16]. Empagliflozin’s metabolism is not regulated by CYPs, but by glucuronidation. No major metabolites are known. Empagliflozin is a substrate for P-glycoprotein and breast cancer resistance protein. The elimination half-life (t_1/2_) of empagliflozin is 7.47 ± 0.97 h [14].

While the clinical efficacy of empagliflozin is well-established, the scientific rationale for rigorously evaluating new formulations in vivo is closely linked to its biopharmaceutical properties. Empagliflozin is classified as a BCS Class III drug, characterized by high solubility but low intestinal permeability [12]. For BCS Class III compounds, the rate and extent of absorption are primarily permeability-limited; thus, formulation-related factors, including excipient composition and manufacturing processes, may influence drug absorption and systemic exposure. Consequently, in vitro dissolution profiles may not fully predict in vivo performance, supporting the need for well-designed human bioequivalence (BE) studies. This is particularly relevant in the context of generic drug development, where demonstration of therapeutic equivalence is essential to ensure interchangeability, quality, and patient safety. Furthermore, given that SGLT2 inhibitors are now foundational therapies for cardiorenal protection in T2D [7], ensuring comparable pharmacokinetic performance between formulations is important to maintain their established therapeutic effects. Finally, despite the high burden of T2D in Mexico [4], there is limited pharmacokinetic evidence regarding empagliflozin formulations in Latin American populations. Therefore, this study aimed to scientifically validate the pharmacokinetic performance and establish the bioequivalence of a new 25 mg empagliflozin immediate-release (IR) formulation in healthy Mexican subjects, contributing region-specific data within a rigorous biopharmaceutical framework, and supporting the availability of high-quality generic alternatives in the local healthcare system.

## 2. Results

### 2.1. In Vitro Dissolution Profiles

**Figure 1:**
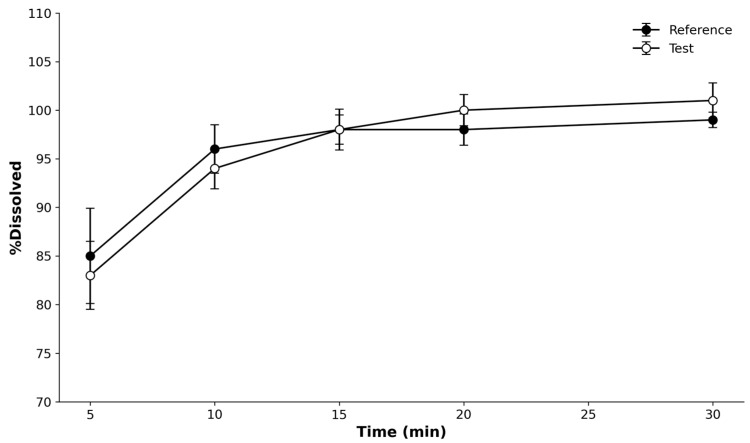
Mean ± SD comparative in vitro dissolution profiles of test (white circle) and reference (black circle) formulations.

The comparative dissolution profiles of the test and reference formulations in phosphate buffer pH 6.8 are shown in Figure 1. Both formulations exhibited very rapid and similar dissolution behavior, achieving more than 85% dissolved drug within 15 min. According to current regulatory criteria, dissolution profiles meeting this condition are considered similar without requiring f2 comparison. Nevertheless, the calculated similarity factor was f2 = 85 [17]. These findings were consistent with the observed in vivo bioequivalence between formulations.

### 2.2. Demographic Characteristics

**Figure 2:**
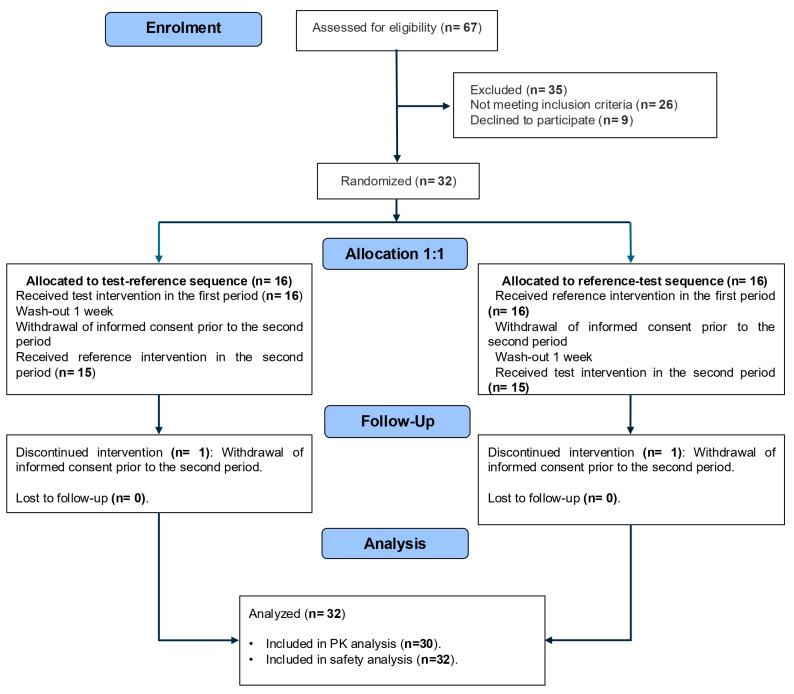
Study flowchart. n: number of subjects; PK: pharmacokinetic.

A total of 32 subjects were enrolled in this study, 30 of whom completed the full protocol (Figure 2). The demographic characteristics of the study population, including age, gender, height, weight and body mass index (BMI), are summarized in Table 1. The total study population consisted of both male and female subjects with a mean ± SD age of 25.10 ± 7.80 years, a height of 1.68 ± 0.08 m, a weight of 66.50 ± 10.30 kg, and a body mass index of 23.40 [2.29] kg/m^2^.

### 2.3. Pharmacokinetics

**Figure 3:**
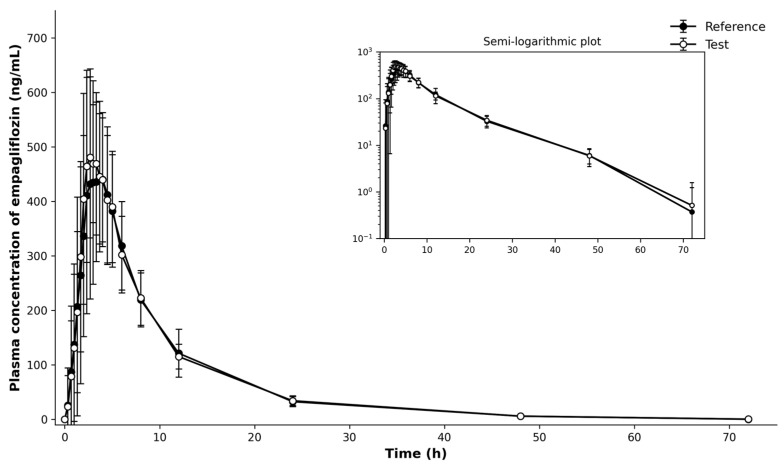
Mean ± SD plasma concentration–time profile of empagliflozin after a single oral dosing of 25 mg tablet. Reference (black circle), test (white circle).

The mean plasma ± standard deviation (SD) concentration–time profiles for empagliflozin following oral administration are shown in Figure 3. The pharmacokinetic parameters (C_max_, t_max_, AUC_0–t_, AUC _0–∞_, and t_½_) for both the test and reference formulations are shown in Table 2. Pharmacokinetic parameters: C_max_, t_max_, AUC_0–t_, AUC _0–∞_, and t_½_ of empagliflozin for test and reference formulation, expressed as mean ± SD, were 578.28 ± 125.60 ng/mL, 2.72 ± 0.85 h, 4370.88 ± 769.50 ngh/mL, 4423.93 ± 776.02 ngh/mL, 7.62 ± 0.83 h, and 593.99 ± 156.78 ng/mL, 2.86 ± 1.00 h, 4313.24 ± 885.02 ngh/mL, 4368.04 ± 887.75 ngh/mL, and 7.61 ± 0.68 h, respectively.

The bioequivalence between both formulations of empagliflozin was evaluated using the geometric mean ratio, and the 90% confidence interval (CI) of the test/reference ratios for log-transformed C_max_, AUC_0–t_, and AUC_0–∞_. The 90% CI for log-transformed C_max_, AUC_0–t_ and AUC_0–∞_ for empagliflozin were 92.72% to 104.22%, 98.77% to 104.77% and 98.73% to 104.63%, respectively (Table 3). All 90% CI of the geometric mean ratios of the pharmacokinetics parameters fell within the pre-specified bioequivalence acceptance limits (80–125%).

### 2.4. Safety and Tolerability Analysis

**Figure 4:**
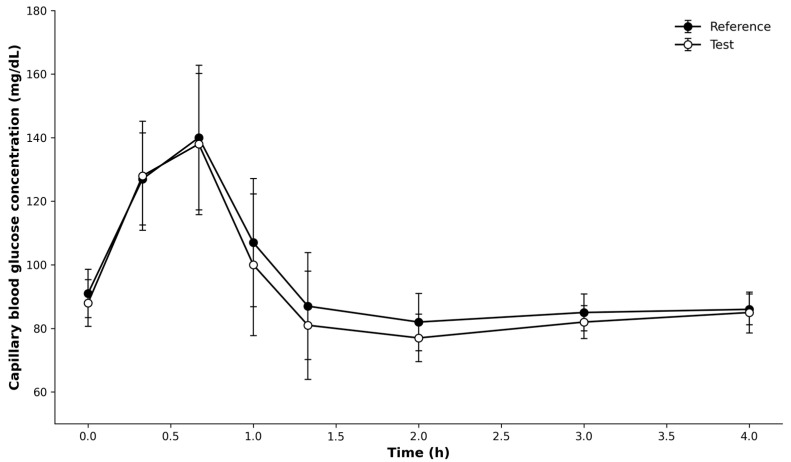
Mean ± SD capillary blood glucose concentration–time profile of empagliflozin after a single oral dosing of 25 mg tablet. Reference (black circle), test (white circle).

During the study, a total of 42 adverse events (AEs) were identified in 14 out of 32 subjects (43.8%). Of the total AEs, 13 AEs (31.0%) were associated with the test formulation (experienced by eight subjects), while 29 (69.0%) occurred with the reference drug product (experienced by 10 subjects). Regarding gender, 13 AEs (31.0%) occurred in men and 29 (69%) in women. In terms of causality, 24 AEs (57.1%) were assessed as unlikely to be related to the study drug, while 18 (42.9%) were considered possibly related to the study drug. A total of 28 (67%) of the events were unexpected, and 14 (33%) were expected according to the study drug. The most frequent events were headache, nausea, and dizziness (Table 4). None of the adverse events were serious, and all were resolved before study close-out. Hypoglycemia (<75 mg/dL) risk was evaluated by capillary plasma glucose measurements for up to 4 h post-dose. Hypoglycemia was observed in 10% and 20% of the test and reference populations, respectively (Figure 4).

## 3. Discussion

Empagliflozin is a potent, selective sodium–glucose cotransporter 2 (SGLT2) inhibitor with a mechanism of action that is independent of β-cell function and insulin sensitivity. SGLT2 inhibitors exert their pharmacodynamic effects by inhibiting renal glucose reabsorption, thereby increasing urinary glucose excretion. These agents carry a low risk of hypoglycemia and do not induce β-cell exhaustion or overstimulation. Consequently, SGLT2 inhibitors represent a viable therapeutic option at any stage of type 2 diabetes management, whether as monotherapy or as an adjunct to other pharmacological interventions [18,19].

The study was designed, according to international guidelines [20] and Mexican regulatory requirements, to evaluate the bioequivalence of 25 mg immediate-release empagliflozin tablets in the fasting state. The study enrolled 32 male and female subjects. According to the initial sample size estimation, this cohort provided sufficient statistical power to demonstrate bioequivalence between the two formulations with a 90% confidence interval, ensuring that the study was adequately powered to detect clinically relevant differences.

The median t_max_ values for the test and reference formulations were 2.72 ± 0.85 h and 2.86 ± 1.00 h, respectively, following a single oral dose of 25 mg empagliflozin in healthy Mexican subjects under fasting conditions. These values indicate a faster absorption rate compared to those reported in Jordanian [21] (4.75 h ± 1.619 h), Chinese [22] (4.0 h) and Venezuelan [14] (3.50 h) populations. Conversely, the absorption was slower than that observed in Korean subjects [23] (1.50 h). Similarly, the mean C_max_ observed in our study (>500 ng/mL) was higher than the approximately 339 ng/mL previously reported in a Venezuelan population [14]. Since the pharmacokinetic profiles compared across these different populations were all evaluated under fasting conditions, the observed variability is unlikely to be related to food effects. Instead, this faster absorption rate and higher peak exposure could be attributed to inter-ethnic variations in gastrointestinal physiology, such as differences in gastric emptying time. Furthermore, because empagliflozin is a BCS Class III [12] drug whose absorption is permeability-limited and acts as a substrate for intestinal efflux transporters like P-glycoprotein, genetic polymorphisms affecting transporter expression (such as those in the ABCB1 gene) among different ethnic groups may significantly contribute to these specific pharmacokinetic variations [24]. The mean value of AUC_0–∞_ was 4368.04 ± 887.75 ng*h/mL in healthy Mexican subjects, which is notably higher than the values reported for Korean [23] and Chinese [22] subjects (3064.17 ± 522.99 ng*h/mL, and 3113.27 ± 508.57 ng*h/mL, respectively), and even than in another Latin population [14] (Venezuelan; 3409.2551 ± 716.71756 ng*h/mL), but lower than the values reported in Jordanian subjects [21] (5270.50 ± 1390.52 ng*h/mL). As shown in Table 2, the mean ± SD t_1/2_ of empagliflozin was 7.62 ± 0.83 h and 7.61 ± 0.68 h for test and reference under fasting conditions, respectively. Therefore, the 7-day washout period was appropriate for the present study, and the carryover effect was not observed. These values were similar to the reported ones in the Venezuelan population [14]; 7.47 ± 0.97 h, shorter that the reported values in Korean [23] and Chinese [22] subjects, 8.59 ± 1.52 h and 8.93 ± 3.66 h, and lower than the reported values in Jordanian subjects [21], 4.00 ± 1.12 h. The observed differences in the rate and extent of absorption in this study might be due to the ethnic differences between study populations.

The 90% confidence interval (CI) for the geometric mean ratios of the primary pharmacokinetic parameters (C_max_ and AUC_0–t_) were entirely within the established bioequivalence acceptance range (80.00–125.00%), as per the regulatory requirements for bioequivalence set by the Mexican Ministry of Health (COFEPRIS) [17]. Furthermore, the ANOVA performed on the log-transformed parameters (C_max_ and AUC_0–t_) revealed no significant effects for formulation, period or sequence.

All adverse events reported during the study were classified as non-serious and mild in intensity, requiring no pharmacological intervention. Although empagliflozin is known to exert a mild antihypertensive effect in chronic use, the difference in acute hypotensive adverse events observed following a single dose (five in the reference group versus 0 in the test group) is likely attributable to random inter-individual variability in healthy normotensive subjects, particularly given the near-identical AUC and C_max_ values between both formulations. All subjects recovered completely without sequelae or further complications. The safety and tolerability profile observed in this study was comparable to that reported in previous single-dose bioequivalence studies of 25 mg empagliflozin.

## 4. Materials and Methods

### 4.1. Drug Products

The test formulation was an immediate-release (IR) tablet containing Empagliflozin 25 mg (Batch No. 73188, expiry date February 2026), manufactured by Farmacéutica Paraguaya S.A. for Asofarma de México, S.A. de C.V (Ciudad de México, Mexico). The reference formulation was Jardiance^®^, an IR tablet containing Empagliflozin 25 mg (Batch No. F42214, expiry date May 2026), manufactured by Boehringer Ingelheim Pharmaceutical Inc. (Ridgefield, CT, USA).

### 4.2. In Vitro Dissolution Studies

Comparative in vitro dissolution profiles of the test and reference formulations were evaluated in phosphate buffer pH 6.8. Dissolution testing was performed using Elite 8 dissolution tester (HANSON RESEARCH, Chatsworth, CA, USA) at 75 rpm in 900 mL of dissolution medium maintained at 37.0 ± 0.5 °C. Samples of 10 mL were collected at 5, 10, 15, 20, and 30 min from 12 dosage units of each formulation, without medium replacement. Empagliflozin dissolved in the samples was quantified by HPLC-UV at 223 nm. The dissolution conditions were selected considering general recommendations described in FDA dissolution guidance documents and the Mexican Pharmacopeia (FEUM 13.0), and the study was conducted following the criteria established in NOM-177-SSA1-2013 for comparative dissolution profile studies used in interchangeability assessments [17]. Similarity between dissolution profiles was assessed using the similarity factor (f2).

### 4.3. Study Design

The study design was a randomized, open-label, two-way, crossover, single-dose, prospective, longitudinal study, with a 7-day washout period before next dosing, to compare the pharmacokinetic profile (C_max_ and AUC_0–t_) of two 25 mg empagliflozin tablet formulations in thirty-two (32) healthy Mexican adult volunteers aged 18 to 55 years, under fasting condition.

The trial was conducted at the third-party research site (a contract research organization also identified as CRO) FS Scientia Pharma, S.A de C.V. [San Luis Potosí, Mexico]. Volunteers were submitted for a 36 h confinement period, admitted to the investigational site from around 16:00 h on day 0 until around 8:00 AM on day 2, for drug dosing, safety and tolerability assessment, and pharmacokinetic blood sampling. Included subjects received a 25 mg empagliflozin tablet with 250 mL of 10% glucose solution (Merck, Naucalpan de Juárez, México) at room temperature under fasting conditions, as part of the hypoglycemia risk management measures required by the national regulatory authority (COFEPRIS), consistent with regulatory recommendations to monitor and prevent hypoglycemia during such studies.

Subjects received a standardized 3000 kcal diet during confinement. Following a mandatory overnight pre-dose fasting period of at least 10 h, the study drug was administered, with water restricted until 4 h post-dose. Pharmacokinetic sampling and vital signs were assessed at scheduled intervals. Subjects were discharged after the initial confinement period, returning at 48.00 and 72.00 h post-dose for further blood collection. Adverse events were rigorously recorded and categorized by duration, severity, causality, and outcome. All participants remained under close medical supervision throughout the study duration.

### 4.4. Study Population

All subjects provided a written informed consent form before the carrying out of any protocol-related procedures or assessments. Moreover, 18–55 year-old healthy male or female volunteers were enrolled. A Body Mass Index [BMI] in the range 18–27 kg/m^2^ was required. Clinical health status was determined at screening visit with the collection of the clinical record, physical examination, 12-lead ECG [electrocardiogram], complete blood cell count, blood chemistry, blood pregnancy test, urinalysis and urine drug test. Additionally, a urine pregnancy test and a breath alcohol test, were performed on baseline. Female subjects of childbearing potential were required to use a highly effective method of contraception during the study period. Key exclusion criteria included pregnancy or lactation, history or evidence of drug or alcohol abuse, clinically significant abnormalities in medical history or laboratory tests, history of significant gastrointestinal surgery or disease, 14-day prior consumption of prescription or over-the-counter drugs and grapefruit or caffeine/xanthine consumption within 10 h before the dosing. All participants were registered on the COFEPRIS volunteer participant database according with the Mexican regulation.

### 4.5. Sample Size

The sample size was determined in accordance with the Mexican regulation NOM-177-SSA1-2013, which establishes that the number of subjects in a bioequivalence study must be based on an appropriate calculation using the intra-subject coefficient of variation (CV_intra_) of the pharmacokinetic parameter with the highest variability (C_max_, AUC_0–t_, or AUC_0–∞_) [17].

Sample size estimation was performed using R software 4.1.2 version (Rcmdr package, 2021) under a two one-sided tests (TOST) procedure for a 2 × 2 crossover design [25]. A significance level (α) of 0.05, a minimum statistical power of 90%, and an assumed test/reference ratio of 0.95 were considered in the calculation.

The intra-subject variability was obtained from the study reported by Hailat et al. (2022), which evaluated the bioequivalence of two 10 mg empagliflozin tablets under fasting conditions in healthy subjects [21]. The highest CV_intra_ was observed for C_max_ (21.41658%), derived from the reported 90% confidence interval (95.69–116.99%). Based on this variability, the minimum required sample size to demonstrate bioequivalence within the 80.00–125.00% acceptance range was estimated to be 28 subjects. To account for potential dropouts or withdrawals, four additional subjects were included, resulting in a total planned sample size of 32 subjects.

### 4.6. Pharmacokinetic Assessment and Analytical Method

Blood samples (6 mL) were collected at 0 h [pre-dose] and at 0.33, 0.67, 1.00, 1.33, 1.67, 2.00, 2.33, 2.67, 3.00, 3.33, 3.67, 4.00, 4.50, 5.00, 6.00, 8.00, 12.00, 24.00, 48.00 and 72.00 h post-dose. Samples were collected in heparinized tubes and centrifuged at 3500 rpm for 10 min at 4 °C. The resulting plasma was transferred into labeled cryovials and stored immediately at −80 ± 10 °C until analysis.

To maintain blinding of the analyst responsible for quantifying empagliflozin plasma concentrations, study samples were coded prior to analysis. The samples were sent to a third-party analytical laboratory (Axis Clinicals Latina, S.A. de C.V.), which was responsible for the bioanalytical determination. Shipment was conducted at −80 °C (±10 °C) on dry ice with temperature monitoring to ensure sample integrity.

Plasma concentrations of empagliflozin were analyzed using a validated high-performance liquid chromatography–tandem mass spectrometry (LC–MS/MS) method developed in accordance with Good Laboratory Practice (GLP) principles. Detailed bioanalytical validation data, including selectivity, precision, accuracy, recovery, matrix effect, and stability assessments, are provided in the Supplementary Materials (Supplementary Material S1).

Sample preparation was performed using a solid-phase extraction (SPE) procedure. Briefly, plasma samples were spiked with 50 µL of the internal standard (empagliflozin-d4), followed by the addition of 500 µL of ammonium hydroxide solution (2%) as a pretreatment step. The samples were vortex-mixed for 10 s and loaded onto pre-conditioned SPE cartridges (Strata-X), previously activated with 1 mL of 100% methanol and equilibrated with 1 mL of deionized water. The cartridges were washed with 1 mL of 10% methanol to remove interfering matrix components, and the analyte was subsequently eluted using 0.5 mL of mobile phase. The eluate was collected and transferred into autosampler vials for LC–MS/MS analysis.

Calibration standards were prepared in human plasma over a concentration range of 2.00–807.21 ng/mL, and quality control (QC) samples were prepared at concentrations of 5.98, 259.90, and 615.89 ng/mL. The calibration curve was constructed using linear regression with a 1/x^2^ weighting factor. Linearity was demonstrated with a correlation coefficient (r) of 0.99904.

Chromatographic separation was achieved on a Prominence LC-20AD SIL-20AC HPLC system (SHIMADZU, Kioto, Japan) equipped with a Viva C8 column 5 µm, 50 × 4.6 mm (RESTEK, Bellefonte, PA, USA) maintained at 40 °C. The mobile phase consisted of methanol: acetonitrile:15 mM ammonium formate (70:5:25, *v*/*v*/*v*), at a flow rate of 0.9 mL/min under isocratic conditions, with a run time of 3.5 min. The autosampler temperature was maintained at 5 °C.

Detection was performed using an QTRAP 4500 triple quadrupole mass spectrometer (ABSCIEX, Woodlands, North Region, Singapore) equipped with a turboIonSpray source operating in positive electrospray ionization mode (ESI+) under multiple reaction monitoring (MRM). The monitored mass transitions were *m*/*z* 468.3 → 355.3 for empagliflozin and *m*/*z* 472.2 → 359.2 for the IS. Data acquisition and integration were performed using Analyst^®^ software version 1.6.2, and study sample concentrations were calculated automatically using the validated chromatographic system.

### 4.7. Tolerability and Safety Assessments

Tolerability was evaluated based on the incidence and severity of the adverse events, physical examination [before dosing and prior to discharge], and serial vital sign monitoring [at baseline and at 1.00, 3.00, 8.00, 12.00, 24.00, 48.00 and 72.00 h post-dose]. To assess the risk of hypoglycemia, capillary plasma glucose levels were measured at 0, 0.33, 0.67, 1.00, 1.33, 2.00, 3.00, and 4.00 h post-dose (Figure 4). In the event of hypoglycemia, a 50% glucose solution was administered via intravenous bolus until normoglycemia was restored.

### 4.8. Statistical Analysis

Descriptive statistics were used to summarize demographic data and assess the homogeneity of the study population. The per-protocol (PP) population, defined as all subjects with evaluable pharmacokinetic data, was included in the PK analysis. The following pharmacokinetic parameters were determined using a non-compartmental analysis (NCA): maximum plasma concentration (C_max_), time to reach C_max_ (t_max_), area under the plasma concentration–time curve from time zero to the last measurable concentration (AUC_0–t_) and extrapolated to infinity (AUC_0–∞_), the terminal elimination rate constant (ke), and the plasma elimination half-life (t_1/2_). Pharmacokinetic parameters were calculated using Phoenix^®^ WinNonlin^®^ version 8.4 software (Certara L.P., Princeton, NJ, USA).

Statistical analysis for bioequivalence assessment was performed using Schuirmann’s two one-sided *t*-test (TOST) for test/reference geometric means ratios of C_max_ and AUC, applying the conventional acceptance criteria of 80–125%. Its 90% confidence intervals were calculated following NOM-177-SSA1-2013 regulation [17].

The intention-to-treat (ITT) population, defined as all subjects who received at least one dose of the study drug, was used for tolerability and safety assessments. Safety evaluation was performed using descriptive statistics and encompassed all observed adverse events.

## 5. Conclusions

The 90% confidence intervals for the geometric mean ratios of the primary pharmacokinetic parameters (C_max_ and AUC_0–t_) fell entirely within the pre-defined regulatory range of 80.00–125.00%. These results conclusively demonstrate bioequivalence between the test and reference empagliflozin formulations. Furthermore, both treatments were well-tolerated, exhibiting an excellent safety profile with no serious adverse events. Consequently, the test formulation represents a therapeutically interchangeable option for improving glycemic control in patients requiring SGLT2 inhibitor therapy.

## Figures and Tables

**Table 1:** Demographic baseline characteristics of study subjects.

Variable	General Population n = 32	Female n = 18	Male n = 14
(%)		56.25	43.75
Age (years) mean ± SD	25.10 ± 7.80	27.89 ± 9.31	25.07 ± 3.20
Weight (kg) mean ± SD	66.50 ± 10.30	57.54 ± 7.35	69.50 ± 9.42
Height (m) mean ± SD	1.68 ± 0.08	1.56 ± 0.06	1.71 ± 0.08
BMI (kg/m^2^) mean ± SD	23.40 ± 2.29	23.38 ± 2.51	23.80 ± 2.58

%: percentage; BMI: Body Mass Index; n: number of subjects; SD: standard deviation.

**Table 2:** Pharmacokinetic parameters of empagliflozin after test and reference dosing. Data are given as mean ± SD.

Parameter	Empagliflozin	
	Test	Reference
C_max_ (ng/mL)	578.28 ± 125.60	593.99 ± 156.78
t_max_ (h)	2.72 ± 0.85	2.86 ± 1.00
AUC_0–t_ (ngh/mL)	4370.88 ± 769.50	4313.24 ± 885.02
AUC_0–∞_ (ngh/mL)	4423.93 ± 776.02	4368.04 ± 887.75
t_½_ (h)	7.62 ± 0.83	7.61 ± 0.68

AUC_0–t_: area under the plasma concentration curve until the last measurable concentration; AUC_0–∞_: area under the plasma concentration curve extrapolated to infinite time; C_max_: maximum plasma concentration; t_max_: time until Cmax is reached; t_1⁄2_: terminal elimination half-life.

**Table 3:** Geometric mean ratios (90% CI) of pharmacokinetic parameters of empagliflozin dosing.

Parameter	Geometric Mean Ratio (%)	90% CI		Schuirmann’s TOST		Power
		LL	UL	*p* < 80%	*p* > 125%	
C_max_ (ng/mL)	98.30	92.72	104.22	0.000001	0.000000	0.99998
AUC_0–t_ (ngh/mL)	101.72	98.77	104.77	0.000000	0.000000	1.00000
AUC_0–∞_ (ngh/mL)	101.64	98.73	104.63	0.000000	0.000000	1.00000

%: percentage; AUC_0–t_: area under the plasma concentration curve until the last measurable concentration; AUC_0–∞_: area under the plasma concentration curve extrapolated to infinite time; CI: confidence interval; C_max_: maximum plasma concentration; LL: lower limit; UL: upper limit.

**Table 4:** Summary of adverse events.

Variable	Test	Reference	Overall
Number of AEs/n			
Overall	13	29	42
Study drug-related	6/13	8/29	14/42
Type of AEs			
Headache	4	6	10
Dizziness	2	3	5
Nausea	3	6	9
Tachycardia	1	1	2
Rhinorrhea	2	0	2
Sore throat	1	1	2
Hypotension	0	5	5
Chills	0	1	1
Fever	0	1	1
Cough	0	1	1
Bradycardia	0	1	1
Diaphoresis	0	1	1
General weakness	0	1	1
Pallor of skin	0	1	1
TOTAL	13	29	42

AEs, adverse events; n, number of AEs.

## Data Availability

The data presented in this study are available upon request from the corresponding author due to confidentiality reasons.

## Supplementary Materials

The following supporting information can be downloaded at: https://www.mdpi.com/article/10.3390/ph19060842/s1. Supplementary Material S1: Bioanalytical Method Validation.

## Abbreviations

The following abbreviations are used in this manuscript:

ADA	American Diabetes Association
AEs	Adverse events
AUC	Area under the curve
BCS	Biopharmaceutical Classification System
BMI	Body mass index
CI	Confidence interval
C_max_	Peak plasma concentration
COFEPRIS	Mexican Ministry of Health
CRO	Contract research organization
ECG	Electrocardiogram
IR	Immediate release
ITT	Intention-to-treat
Ke	Terminal elimination rate constant
LL	Lower limit
NCA	Non-compartmental analysis
PK	Pharmacokinetics
PP	Per-protocol
RLD	Reference listed drug
SGLT2	Sodium–glucose cotransporter 2
SD	Standard deviation
t_1/2_	Elimination half-life
t_max_	Time to reach peak plasma concentration
T2D	Type 2 diabetes
UL	Upper limit
