# Supplementary material for: Bioequivalence of Two Empagliflozin 25 mg Immediate-Release Tablet Formulations Under Fasting Conditions in Healthy Mexican Subjects
Source: Pharmaceuticals (Basel). 2026 May 28;19(6):842. doi: 10.3390/ph19060842 (PMC13304527; doi:10.3390/ph19060842)
Supplement: Supplementary file 1 [file pharmaceuticals-19-00842-s001.zip › pharmaceuticals-4297991-supplementary.pdf]

1. Supplementary material: Bioanalytical Method Validation

Validation of an Analytical Method for the Determination of Empagliflozin in Human K_2EDTA Plasma by LC–MS/MS (Information extracted and summarized from the bioanalytical validation report)

1.1 Objective and regulatory framework

The aim of this study was the full validation of an analytical method for the determination of empagliflozin in human K_2EDTA plasma.

The bioanalytical method validation was conducted in accordance with the applicable regulatory requirements for bioequivalence studies in Mexico (NOM-177-SSA1-2013). The validation approach is consistent with internationally accepted principles described in regulatory guidelines, including those from the European Medicines Agency (EMA) and the U.S. Food and Drug Administration (FDA), as well as Good Laboratory Practice (GLP) standards.

1.2 Summary of the analytical method

Empagliflozin concentrations in human K_2EDTA plasma were determined using a validated LC-MS/MS method with empagliflozin-d4 as the internal standard (IS). Sample preparation was performed by solid-phase extraction (SPE). Quantification was based on analyte-to-internal standard peak area ratios, and calibration curves were constructed using least-squares linear regression with a $1/x^2$ weighting factor.

1.2.1 Linearity and calibration curve

Calibration standards were prepared in human plasma over the concentration range of 2.00-807.21 ng/mL. Calibration curves were constructed using analyte-to-internal standard peak area ratios across the studied concentration range.

The calibration model showed excellent linearity, with a mean correlation coefficient (r) of 0.99904.

Table S1. Measured concentrations and variability across three independent calibration runs for the evaluation of method linearity of empagliflozin in human plasma.

**Run**	**Nominal concentration** (ng/mL)							
	**2.00**	**4.00**	**20.00**	**88.19**	**176.37**	**352.75**	**629.91**	**807.57**
1	1.98	4.08	20.11	89.94	173.26	342.39	668.81	767.77
2	1.95	4.18	20.00	90.81	174.30	346.30	637.44	780.35
3	2.04	3.88	19.44	87.59	177.95	356.45	630.50	829.26
**Mean**	1.990	4.047	19.850	89.447	175.170	348.380	645.583	792.460

**SD**	0.0458	0.1528	0.3593	1.6657	2.4631	7.2571	20.4120	32.4845
**%CV**	2.3	3.8	1.8	1.9	1.4	2.1	3.2	4.1

SD: standard deviation; %CV: coefficient of variation (%).

Table S2. Calibration curve parameters for empagliflozin.

**Run**	**Slope (m)**	**Intercept (b)**	**Correlation coefficient (r)**
1	0.9874	1.7152	0.9976
2	0.9806	1.8629	0.9996
3	1.0187	-1.5757	0.9999
**Mean**	0.99558	0.66748	0.99904

Figure S1. Representative calibration curve obtained using analyte-to-internal standard peak area ratios, showing the linear relationship between concentration and response for empagliflozin in human plasma.

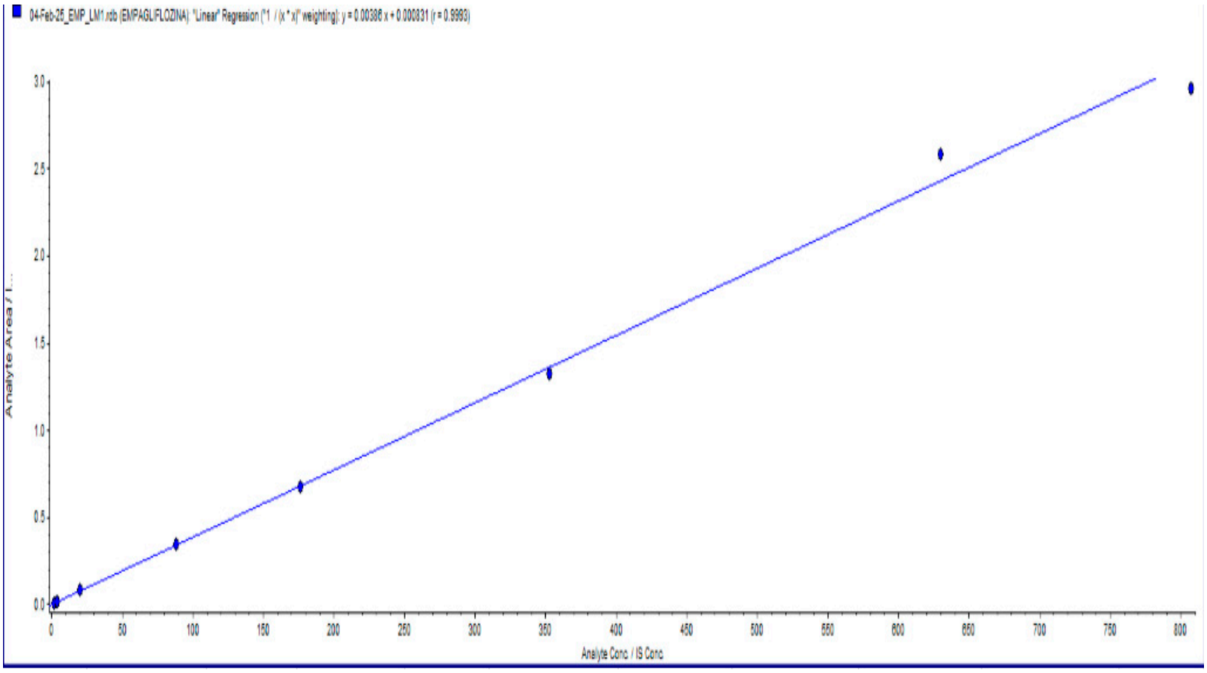

1.2.2 Accuracy and precision

Intra-day and inter-day accuracy and precision were evaluated using quality control (QC) samples at multiple concentration levels.

For repeatability, six replicates of the lower limit of quantification (2.01 ng/mL), as well as plasma quality control (QC) samples at low, medium, and high concentrations (5.98, 260.05, and 616.23 ng/mL), were analyzed in sextuplicate. In addition, six replicates of the highest calibration standard diluted at ratios of 1/2 and 1/4 were evaluated.

For reproducibility, six replicates of the lower limit of quantification (2.01 ng/mL) and plasma quality control samples at low, medium, and high concentrations (5.98, 260.05, and 616.23 ng/mL) were analyzed in sextuplicate, with three determinations performed by each analyst on at least two different days.

The method showed adequate precision, with coefficients of variation (CV) below 15%, and accuracy expressed as absolute deviation (AD) within 15% of nominal concentrations across all QC (quality control) levels. For the lower limit of quantification (LLOQ), both precision (CV ≤ 20%) and accuracy (AD ≤ 20%) were within the predefined acceptance criteria.

1.2.3 Selectivity and matrix effect

Selectivity was evaluated using multiple individual plasma sources, including hemolyzed and lipemic samples, as well as plasma containing commonly co-administered drugs.

No significant interference was observed at the retention times of empagliflozin and the internal standard (IS). Matrix effects were assessed and demonstrated acceptable variability, with normalized matrix factor CV values below 15% at both QC (quality control) levels.

1.2.4 Recovery

Extraction recovery was evaluated at different concentration levels. The method showed consistent recovery across the concentration range for both empagliflozin and the internal standard, with CV values below 15%

1.2.5 Lower limit of quantification (LLOQ)

The LLOQ was established at 2.00 ng/mL, demonstrating acceptable accuracy and precision (CV ≤ 20% and absolute deviation ≤20%), confirming the suitability of the method for quantifying low plasma concentrations.

1.2.6 Stability

Stability of empagliflozin in plasma was evaluated at two concentration levels in human plasma analyzed in sextuplicate (5.98 and 616.23 ng/mL), under various conditions, including:

- Short-term (room temperature) stability (22.57 hours)
- Autosampler stability (5°C, 48.13 hours).
- Freeze–thaw cycles (6 cycles).
- Long-term storage stability.
- Refrigerated processed sample stability (44.83 hours)
- Working solution stability (short- and long-term) (23.98 hours)
- Stock solution stability (short- and long-term) (23.88 hours)
- Whole blood stability. (3.07 hours)

Empagliflozin was found to be stable under all tested conditions, with precision (CV ≤ 15% and accuracy expressed as absolute deviation ≤ 15%) within the predefined acceptance criteria.

1.3 Overall validation performance

The validation results demonstrated that the analytical method met all predefined acceptance criteria for bioanalytical method validation. The method showed adequate linearity, precision, accuracy expressed as absolute deviation, selectivity, recovery, sensitivity, and stability under all evaluated conditions.

Table S3. Summary of bioanalytical method validation results for empagliflozin.

Parameter	Acceptance criteria	Result
Linearity	r ≥ 0.98	0.99904
Precision (intra-day)	CV ≤ 15%	0.9-2.6%
Accuracy (intra-day)	Absolute deviation (AD) ≤15%	1.1-4.3%
Precision (inter-day)	CV ≤ 15%	3.8-5.5%
Accuracy (inter-day)	AD ≤15%	2.6-8.1%

Selectivity	No interference at the retention time of the analyte and internal standard	No interference was observed at the retention time of empagliflozin and the IS in blank, hemolyzed, and lipemic plasma, or in the presence of tested drugs (acetaminophen, ibuprofen, ketorolac, ondansetron, loratadine, metformin, salicylic acid, metamizole, loperamide, etonogestrel, levonorgestrel, dimenhydrinate)
LLOQ	CV ≤ 20%, AD ≤20%	2.01 ng/mL (CV 10.7%, AD 6.0)
Carryover	Carryover at the analyte retention time < 20% and carryover at the internal standard (IS) retention time < 5%	0.0% for empagliflozin and 0.0% for empagliflozin-d4
Recovery	CV ≤ 15%	9.9% (empagliflozin), 8.1 % (empaglifozin-d4)
Reinjection reproducibility	CV ≤ 15%, AD ≤ 15%	Stable up to 19.48 h (CV 0.9–3.8%, AD 1.2–6.0%)
Matrix effect	Normalized matrix factor CV ≤ 15% at both QC levels	2.5% (low QC), 2.1% (high QC)
Stability (short-term, room temperature)	CV ≤ 15%, AD ≤ 15%	Stable up to 22.57 h (CV 2.4%-3.2%, AD 2.8%-3.9%)
Stability (autosampler at 5 °C)	CV ≤ 15%, AD ≤ 15%	Stable up to 48.13 h (CV 5.0%-5.7%, AD 1.5%-9.0%)

Freeze–thaw stability (-70°C, 6 cycles)	CV ≤ 15%, AD ≤ 15%	Stable for 6 cycles (CV 2.7%-5.1%, AD 0.8%-6.4%)
Long-term stability	CV ≤ 15%, AD ≤ 15%	Stable up to 29 days (CV 2.9%-5.8%, AD 3.6-4.3%)
Refrigerated processed sample stability (8°C)	CV ≤ 15%, AD ≤ 15%	Stable up to 44.83 h (CV 1.2-7.2%, AD 0.8-5.7%)
Working solution stability (short- and long-term)	AD ≤ 10%	Stable up to 23.98 h (high, short-term), 23.93 h (low, short-term), and 29 days (high and low,long-term)<br>CV 1.1–1.7% and AD 2.3–2.9% (high, short-term); CV 2.2–3.9% and AD 4.7–5.7% (low, short-term); CV 1.6–1.7% and AD 6.8–9.0% (high, long-term); CV 3.1–3.2% and AD 0.0–0.5% (low, long-term)
Stock solution stability (short- and long-term)	AD ≤ 10%	Stable up to 23.88 h (short-term) and 29 days (long-term); CV 0.8–1.5% and AD 1.6–2.1% (short-term); CV 0.9–2.0% and AD 0.3–1.0% (long-term)
Whole blood stability	CV ≤ 15%, AD ≤ 15%	Stable up to 3.07 h (CV 1.9–2.5%, AD 0.5–2.9%)
